# Utilizing Tumor and Plasma Liquid Biopsy in Treatment Decision Making for an Estrogen Receptor-Positive Advanced Breast Cancer Patient

**DOI:** 10.7759/cureus.1408

**Published:** 2017-06-29

**Authors:** Bing Xu, Amy Krie, Pradip De, Casey Williams, Rachel Elsey, Jessica Klein, Brian Leyland-Jones

**Affiliations:** 1 Molecular and Experimental Medicine, Avera Cancer Institute

**Keywords:** er+ breast cancer, precision ocology, circulating tumor dna, molecular profiling

## Abstract

Breast cancer affects 12% of females in the United States and is the leading cause of cancer death in the female population. Personalized therapy is being used in clinical practice to treat breast cancer based on tumor molecular profiling, which can be obtained from tissue biopsy or plasma liquid biopsy as circulating tumor deoxyribonucleic acid (ctDNA). The available ctDNA tests provide a non-invasive way to monitor the cancer genome in a real-time manner.

In this case report, a 38-year-old female with recurrent estrogen receptor (ER) positive breast cancer is treated with letrozole, everolimus, and palbociclib. The drugs target the hormonal signaling pathway, phosphoinositide 3-kinase (PI3K)-RAC-alpha serine/threonine-protein kinase (AKT) pathway, and cyclin D1 (CCND1)-CDK4/6 pathway, based on the patient’s estrogen-receptor-positive (ER+) disease and phosphatidylinositol -4,5-bisphosphate 3-kinase catalytic subunit alpha (*PIK3CA*) mutation, as well as *PIK3CA* and *CCND1 *amplification. After 11 months of treatment, retinoblastoma protein transcriptional corepressor 1 (*RB1*) mutation was caught in ctDNA, which suggests an acquired resistance to palbociclib. Pazopanib was then used instead of palbociclib, targeting the* fibroblast growth factor 3/4/19 (FGF3/4/19)* amplification that was initially observed in her molecular profiling. Trametinib was also suggested recently due to the increasing allele frequency of B-Raf proto-oncogene, serine/threonine kinase (*BRAF*) mutation in ctDNA, following the treatment of letrozole + everolimus + pazopanib. The patient has no evidence of disease after five months of treatment initiation and has remained disease-free for over 16 months.

In conclusion, the analysis of ctDNA is an effective way to monitor the real-time changes in a patient's tumor genome, which is a great supplement to the molecular profile from the tissue biopsy. The combination of these two tests provides an efficient strategy to make more informed treatment decisions, which greatly adapt along disease development.

## Introduction

Breast cancer is a leading cause of cancer death in females worldwide [1]. According to the United States breast cancer statistics, about 12% of women are affected by the disease in America. Among breast cancer patients, over 70% of them have estrogen receptor (ER) positive disease. After decades of investigations, the research communities have a far greater understanding of the disease at the molecular level, especially with the identification of genetic alterations related to the development of breast cancer. With the continuous development of sequencing techniques in recent years, personalized treatment plans are in practice based on molecular profiling for the specific patient.

Among breast cancer patients, molecular profiling is normally performed using a biopsy from either primary or metastatic tumor tissue. However, the genetic changes of cancer are not stable but dynamic. It has been known that cancer is a genetic disease. This results in heterogeneous tumors that change continuously throughout the course of the disease. Treatment plans need to be adjusted according to the changes. But in practice, obtaining biopsy from patients frequently to update their molecular profiles is not practical.

With the development of the liquid biopsy and sequencing using circulating tumor deoxyribonucleic acid (ctDNA) from plasma samples, a new approach is providing the opportunity for closer monitoring of disease development. Plasma ctDNA is obtained in a non-invasive way as a blood draw, which allows clinicians to have a real-time evaluation of the tumor burden, discover emerging genetic alterations and change treatment regimen in a timely manner.

In this case of ER-positive metastatic ductal carcinoma of the breast, we found dynamic changes in ctDNA, including changes in allele frequency of existing mutations and presenting of new genetic alterations. The treatment plan was originally made based on the molecular profile of the tumor biopsy and changed based on ctDNA findings. Based on this strategy, the patient has remained disease free for over 16 months after recurrence. Informed consent statement was obtained for this study.

## Case presentation

A 38-year-old Caucasian female was seen in our clinic in August 2015 with a recurrent disease of the breast in the left supraclavicular lymph node.

The patient was first diagnosed in 2011. The tumor was grade 3 (3/2/3) by Nottingham grade; estrogen receptor (ER) positive, progesterone receptor (PR) positive and human epidermal growth factor receptor 2 (HER2) negative by immunohistochemistry (IHC). Right breast mastectomy and axillary lymph node dissection were performed with left breast prophylactic mastectomy. Final pathology suggested a stage IIB (T2N1aM0) invasive ductal carcinoma according to the Union for International Cancer Control (UICC) Tumor Node Metastasis (TNM) classification for breast cancer. The tumor measured 4.4 cm. Two out of nine lymph nodes were positive for cancer invasion. The patient was treated with dose-dense doxorubicin and cyclophosphamide followed by paclitaxel (AC-T) after surgery and completed post-mastectomy radiation therapy to the right breast in May 2012.

The patient was then maintained on estrogen blocker (tamoxifen) and ovarian suppressor (leuprolide) from May 2012 till December 2014 when leuprolide was stopped because of insurance reasons. The patient continued on tamoxifen.

In June 2015, the patient was found to have fullness in the left supraclavicular area. Positron emission tomography–computed tomography (PET/CT) demonstrated eight small lymph nodes in the left supraclavicular region and one lymph node in the right paratracheal area with fluorodeoxyglucose (FDG) uptake. Biopsy of left supraclavicular lymph node demonstrated poorly differentiated invasive ductal carcinoma consistent with ER positive, PR positive and HER2 negative by fluorescence in situ hybridization (FISH).

In addition, the same biopsy was tested through FoundationOne® for genomic profiling. FoundationOne® is a comprehensive genomic profiling assay, sequencing the coding region of 315 cancer-related genes and introns from 28 genes to a median depth of coverage of 500X. Alterations detected by FoundationOne® are presented in Table 1. DNA repair associated breast cancer 1/2 (*BRCA1/2* ) test from Comprehensive BRACAnalysis® (MYRIAD Genetic Laboratories, Salt Lake City, Utah ) came back with negative results.

**Table 1:** Table representing the genetic alterations from tumor biopsy Ala: Alanine; Arg: Arginine; Asp: Asparagine;Cys: Cysteine; Gly: Glycine; His: Histidine; Leu: Leucine; Phe: Phenylalanine; Pro: Proline; Ser: Serine; Thr: Threonine; Trp: Tryptophan; Tyr: Tyrosine; Val: Valine. *ATM*: Serine-protein kinase ATM; *CCND1*: G1/S-specific cyclin-D1; *EMSY*: BRCA2-interacting transcriptional repressor EMSY; *FANCF*: Fanconi anemia group F protein; *FGF19*: Fibroblast growth factor 19; *FGF3*: Fibroblast growth factor 3; *FGF4*: Fibroblast growth factor 4; *FOXP1*: Forkhead box protein P1; *GPR124*: G-protein coupled receptor 124; *MLL3*: lysine methyltransferase 2C; *MYST3:* MYST histone acetyltransferase (monocytic leukemia) 3; *PALB2*: partner and localizer of BRCA2; *PIK3CA*: phosphatidylinositol-4,5-bisphosphate 3-kinase catalytic subunit alpha; *TP53*: Tumor Protein P53; *ZNF703*: Zinc finger protein 703

Gene	Effect (amino acid change)	Gene	Effect (copy number change, rearrangement and insertion)
PIK3CA	p.His1047Leu	PIK3CA	Amplification
TP53	p.Arg248Trp	CCND1	Amplification
ATM	p.Ser1691Arg	FGF19	Amplification
FANCF	p.Ala186Val	FGF4	Amplification
FOXP1	p.His520Tyr	EMSY	Amplification
GPR124	p.Ser1276Arg	FGF3	Amplification
MLL3	p.Ser836Phe	PALB2	Rearrangement exon 5 -- truncation
MYST3	p.Ala1521Ser	ZNF703	His402_Asp403>ProThrHisLeuGlyGlySerSerCysSerThrCysSerAlaHisAsp

Based on the patient’s disease status, tumor marker (ER/PR/HER2) and genetic testing results, we started the following regimen with letrozole (2.5 mg daily), palbociclib (75mg day one through day 21 of a 28- day cycle) and everolimus (5 mg Monday, Wednesday, Friday). The patient started this regimen in August 2015. A follow-up PET/CT scan in January 2016 suggested the previously noted hypermetabolic lymph nodes in the left neck and in the mediastinum from June 2015 have resolved and no evidence for metastatic disease was detected. With the excellent response to the therapy, the patient was kept on this regimen for over a year. Follow-up scans in April 2016 suggested no evidence of disease as the previous one.

During the treatment, ctDNA was monitored through Guardant360® test in 2016 and FoundationACT® test in 2017. Both Guardant360® and FoundationACT® use plasma DNA. Guardant360® covers the exons of 73 genes. FoundationACT® interrogates a total of 62 genes. All ctDNA results for this patient are presented in Table 2.

**Table 2:** Table representing the plasma circulating tumor deoxyribonucleic acid (ctDNA) and allele frequencies Phe: Phenylalanine; Leu: Leucine; Thr: Threonine; Pro: Proline; Glu: Glutamic acid; Lys: Lysine. *BRAF*: B-Raf proto-oncogene, serine/threonine kinase; *RB1*: RB transcriptional corepressor 1; *NOTCH1*: notch 1. ND: Not detected.

Test Vendor		Guardant360				FoundationAct	
Gene		1/7/16	2/4/16	7/7/16	10/27/16	2/15/17	4/13/17
BRAF	Phe595Leu	0.4	0.1	0.3	0.3	0.34	0.59
RB1	Thr12Pro	ND	ND	1.7	ND	ND	ND
NOTCH1	Glu360Lys	ND	ND	ND	0.1	ND	ND

The first two samples of plasma DNA were drawn in January and February of 2016. The results suggested *BRAF* (p.Phe595Leu) alteration with allele frequencies of 0.4% and 0.1% respectively. In July 2016, *RB1* (p.Thr12Pro) mutation with an allele frequency of 1.7% showed up in plasma ctDNA, together with the *BRAF* mutation. Based on the new *RB1* mutation, palbociclib was switched to pazopanib (200 mg every other day) in September 2016. The ctDNA results from the end of October demonstrated resolution of the *RB1* mutation with the only presence of 0.3% *BRAF* mutation. PET/CT in September and November 2016 suggested no evidence of disease. However, an increase of BRAF mutation in ctDNA was observed. The allele frequencies from February and April 2017 for the *BRAF* mutation were 0.34% and 0.59% respectively. The patient was kept on letrozole, everolimus, and pazopanib until May 2017. Due to the rise of the allele frequency of *BRAF* (p. Phe595Leu) in ctDNA from 0.34% to 0.59%, we are planning to discontinue pazopanib and start trametinib.

The latest scan was obtained for the patient in May 2017. No evidence of disease was detected.

**Figure 1:**
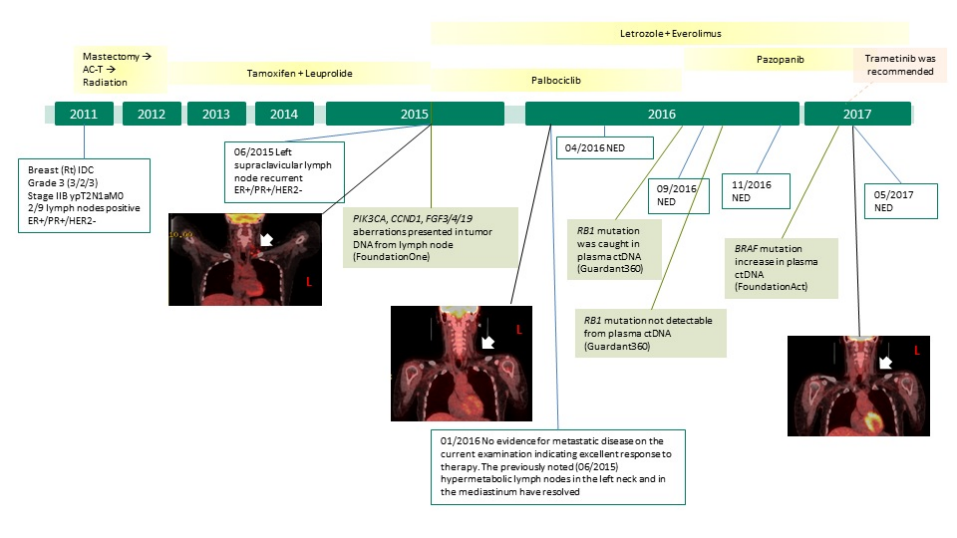
Figure showing the history of present illness and treatment The disease progression and treatment history is presented in this figure. The positron emission tomography–computed tomography (PET/CT) images are showing the recurrent disease in 2015 and clear scans from 2016. Treatment regimens are presented in the yellow boxes and significant genomic findings are in light green boxes.

An illustration of disease progression and treatment for this patient is presented in Figure 1.

## Discussion

In the case described above, after disease recurrence, the next line treatment recommendation was made based on the molecular profile from the recurrent tumor biopsy at the patient’s lymph nodes as well as her tumor marker results.

Since the disease was ER positive, letrozole was selected first in the regimen as an aromatase inhibitor to lower estrogen level. One of the most commonly modified pathways in ER-positive breast cancer is phosphoinositide 3-kinase (PI3K)- RAC-alpha serine/threonine-protein kinase (AKT) pathway. Activation of this pathway is associated with resistance to hormonal therapy, while promising results have been suggested by clinical trials using the combination of endocrine therapy and PI3K-AKT pathway inhibitors [2]. *PIK3CA* encodes the catalytic subunit of PI3K. Both *PIK3CA* amplification and mutation, as detected in this patient are associated with an enhancement of downstream signaling and over-activation of Akt. Akt blocks mammalian target of rapamycin complex 1 (mTORC1) inhibitor tuberous sclerosis proteins 1 and 2 (TSC1/2). Without TSC1/2, mTORC1 is over activated and phosphorylates eukaryotic translation initiation factor 4E-binding protein 1 (4E-BP1) and ribosomal protein S6 kinase (S6K), which increase translation and promotes cell proliferation. Everolimus was added to the regimen as an mTORC1 inhibitor, targeting the *PIK3CA* aberrations found in the patient [3].

Another pathogenic alteration suggested by the patient’s molecular profile is *CCND1* amplification. The vast majority of *CCND1* amplified breast cancer are reported to be ER-positive and associated with poor prognosis [4]. In estrogen-driven breast cancer, oncogenic signaling through estrogen stimulated the CCND1-CDK4/6-dependent retinoblastoma protein (Rb). CCND1-CDK4/6 complex phosphorylates Rb and releases transcription factor E2F (E2F) from the Rb-E2F complex. Once E2F is released, it can switch cells from G1 to S phase and start cell proliferation. This proliferative stimulus was augmented by amplification of *CCND1*. Palbociclib was included in the regimen as a cyclin-dependent kinase 4 and 6 (CDK4/6) inhibitor to block the phosphorylation of Rb and inducing G1 arrest.

However, a good clinical response of palbociclib relies on a wild type, functioning Rb protein. Loss of Rb function was reported in different cases to relate with palbociclib resistance [5-6]. When *RB1* mutation was caught in ctDNA from this patient, palbociclib was taken off from the regimen.

Another potential target in the molecular profile from tumor biopsy was fibroblast growth factor 3, 4 and 19 (*FGF3/4/19*), which indicated an active ancillary angiogenic pathway. Pazopanib was introduced to the treatment plan when palbociclib was stopped, to prevent treatment resistance of palbociclib and further block the activities of the FGFs. Upon the binding of FGFs to the FGF receptors (FGFR), FGFRs forms dimers. The dimerization process activates FGFR kinase activity. Activated FGFR kinase further phosphorylates and activates different pathways, which promotes cell proliferation, survival, and motility [7]. Pazopanib, as a pan-tyrosine kinase inhibitor including FGFR, is proven to be clinical beneficial from different trials for breast cancer [8-9].

Although the patient remained disease free on the treatment, the consistent and rising *BRAF* (p.Phe595Leu) mutation in ctDNA caused concerns. *BRAF* mutations in the kinase domain can result in an elevated kinase activity, which directly phosphorylates and activates serine/threonine protein kinase MEK1/2 (MEK1/2) on the Ras-Raf pathway. The over-activation of this Ras-Raf pathway can further induce a series of Elk-dependent transcription and increase cell growth and proliferation [10]. In order to prevent tumor recurrent from the *BRAF* mutation, trametinib will be started in the next cycle as an MEK1/2 inhibitor to block the Ras-Raf pathway.

**Figure 2:**
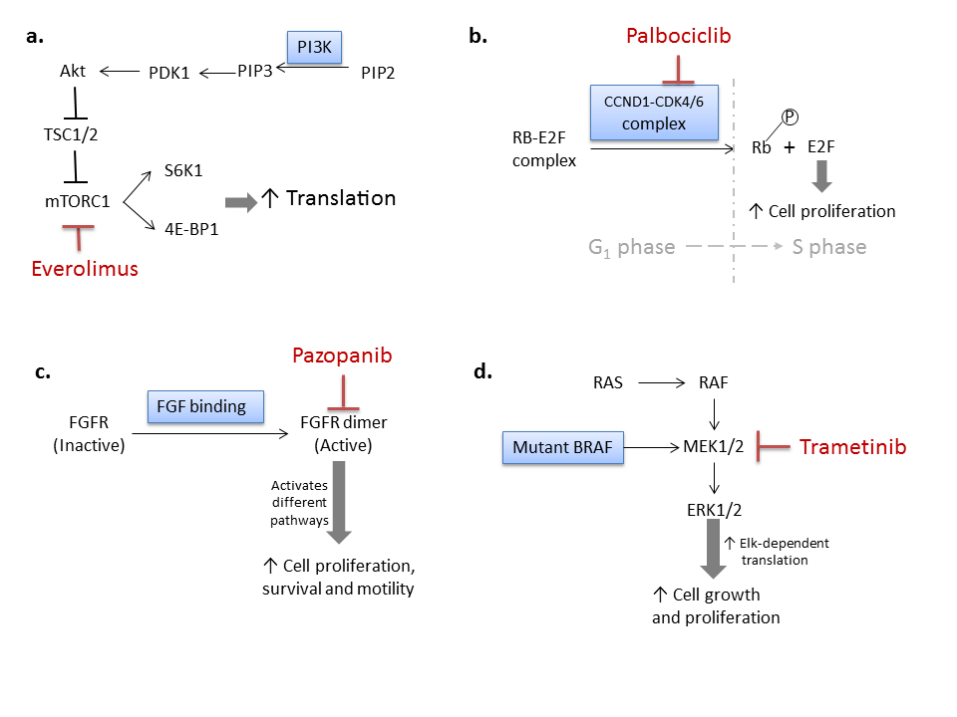
Figure showing the rationale of treatment recommendations Simplified signaling pathways with treatment targets related to the patient are presented in this figure. Molecular aberrations are placed in the blue boxes. Drugs are presented in red. *a) *Everolimus as a mTROC1 inhibitor targets the bisphosphate 3-kinase catalytic subunit alpha (PIK3CA) aberrations. *b)* Cyclin D1​ (*CCND1)* amplification promotes G_1_/S transition through releasing E2F by phosphorylating retinoblastoma protein (Rb). Palbociclib functions to block the G_1_/S transition by blocking cyclin-dependent kinase 4 and 6 (CDK4/6) kinase activity, which relies on wild-type, fully functioning Rb. *c)* When retinoblastoma protein-*RB1* mutation was caught on circulating tumor deoxyribonucleic acid (ctDNA), pazopanib took the place of palbociclib in the regimen, as a fibroblast growth factor (FGFR) inhibitor, targeting the *FGF3/4/19 *amplifications. *d) *Mutant *BRAF* in the kinase domain indicates an elevated RAS-RAF pathway. Trametinib is suggested to block MEK1/2 activity when allele frequency of the *BRAF *kinease domain mutation increased in ctDNA.

The rationale of the regimens is illustrated in Figure 2 and abbreviations are listed under Table 3.

**Table 3:** Table showing the list of abbreviations for reference

List of Abbreviations	
4E-BP1	Eukaryotic translation initiation factor 4E-binding protein 1
AKT	RAC-alpha serine/threonine-protein kinase
ATM	Serine-protein kinase ATM
CCND1	G1/S-specific cyclin-D1
E2F	Transcription factor E2F
EMSY	BRCA2-interacting transcriptional repressor EMSY
FANCF	Fanconi anemia group F protein
FGF19	Fibroblast growth factor 19
FGF3	Fibroblast growth factor 3
FGF4	Fibroblast growth factor 4
FOXP1	Forkhead box protein P1
GPR124	G-protein coupled receptor 124
MEK1/2	serine/threonine protein kinase MEK1/2
MLL3	lysine methyltransferase 2C
mTOR	mammalian target of rapamycin
MYST3	MYST histone acetyltransferase (monocytic leukemia) 3
PALB2	partner and localizer of BRCA2
PIK3CA	phosphatidylinositol-4,5-bisphosphate 3-kinase catalytic subunit alpha
Rb	retinoblastoma protein
RB1	RB transcriptional corepressor 1
S6K	ribosomal protein S6 kinase
TP53	Tumor Protein P53
TSC1/2	Tuberous sclerosis proteins 1 and 2
ZNF703	Zinc finger protein 703

## Conclusions

Based on the initial molecular profile from a tumor biopsy, we are able to restrict the disease progression and no evidence of disease is suggested on PET/CT after five months of targeted therapy. Also with the information provided by a sequence of ctDNA testing, the changes of tumor genome are closely monitored and the treatment regimens are adjusted accordingly in a timely manner. Benefited from both tumor tissue and ctDNA sequencing, a series of personalized treatment plans are made for the patient, aiming at targeting the drivers of the disease precisely and prevent acquired treatment resistance at the best possibility. As a result, the patient is successfully maintained in disease-free status for over 16 months.

We are planning to follow the treatment protocol, re-check ctDNA and imaging every three months and change the regimen when necessary. With this plan, we are confident to maintain the patient in disease-free status without further cancer progression.

## References

[1] Ferlay J, Soerjomataram I, Dikshit R (2015). Cancer incidence and mortality worldwide: Sources, methods and major patterns in GLOBOCAN 2012. International journal of cancer 2015. Int J Cancer.

[2] Paplomata E, O’Regan R (2014). The PI3K/AKT/mTOR pathway in breast cancer: targets, trials and biomarkers. Therapeutic advances in medical. Ther Adv Med Oncol.

[3] Dienstmann R, Rodon J, Serra V (2014). Picking the point of inhibition: A comparative review of PI3K/AKT/mTOR pathway inhibitors. Mol Cancer Ther.

[4] Tobin NP, Bergh J (2012). Analysis of cyclin D1 in breast cancer: A call to arms. Current breast cancer. J Curr Breast Cancer Rep.

[5] Roberts PJ, Bisi JE, Strum JC (2012). Multiple roles of cyclin-dependent kinase 4/6 inhibitors in cancer therapy. J Natl Cancer Inst.

[6] Malorni L, Piazza S, Ciani Y (2016). A gene expression signature of retinoblastoma loss-of-function is a predictive biomarker of resistance to palbociclib in breast cancer cell lines and is prognostic in patients with ER-positive early breast cancer. Oncotarget.

[7] Goetz R, Mohammadi M (2013). Exploring mechanisms of FGF signalling through the lens of structural biology. Nat Rev Mol Cell Biol.

[8] Turner NC, Ro J, Andre F (2015). Palbociclib in hormone-receptor–positive advanced breast cancer. N Engl J Med.

[9] Finn RS, Crown JP, Ettl J (2016). Efficacy and safety of palbociclib in combination with letrozole as first-line treatment of ER-positive, HER2-negative, advanced breast cancer: expanded analyses of subgroups from the randomized pivotal trial PALOMA-1/TRIO-18. Breast Cancer Res.

[10] Davies H, Bignell GR, Cox C (2002). Mutations of the BRAF gene in human cancer. Nature.

